# Protein intake and nutritional programming: metabolic consequences

**DOI:** 10.1186/1824-7288-40-S1-A49

**Published:** 2014-08-11

**Authors:** Elvira Verduci, Benedetta Mariani, Carlotta Lassandro, Alice ReDionigi

**Affiliations:** 1 Department of Pediatrics, San Paolo Hospital, Department of Health Sciences, University of Milan, Milan, Italy

## 

According to recent epidemiological evidence, early protein intakes that exceeds metabolic requirements (>15% of energy) may increase weight gain during infancy and the risk of developing obesity in childhood: the so-called “early protein hypothesis” [1]. A high protein intake, indeed, especially milk’s protein [2,3], may enhance the secretion of insulin and insulin-like growth factor-I (IGF-I), associated with increased weight gain during the first 2 years of life, increase of adipocyte differentiation and adipogenic activity [4]. In a large number of studies increased weight gain in infancy and early adiposity rebound have been associated with the development of later obesity [4]. The presence of a positive strong association between early protein intake and increased weight gain in early childhood has been recently demonstrated in the European Childhood Obesity Project (CHOP): a multicenter, double-blind intervention trial involving both formula fed infants, randomly assigned to receive, during the first year of life, infant and follow-on formulas with different protein content (high or low), and breastfed infants as control group. This trial showed that both weight-for-length and BMI z-scores were significantly higher in the high protein (HP) compared with the low protein (LP) group at 12 and 24 months [5]. Moreover the body composition analysis at 6 months of life showed that weight gain velocity from baseline to 6 months was significantly associated with fat mass, proving that higher early protein intakes may influence adiposity [6]. Concerning metabolic data, HP group compared with LP group showed higher plasma concentrations of branched chain aminoacids, IGF-I and insulin at 6 months. Moreover, IGF-I concentrations have been associated with weight gain in the first 6 months of life [7]. Additionally IGF-I could partly mediate protein-induced kidney growth in healthy children [8]. This phenomenon was observed in the HP group: children at 6 months of life showed a significantly greater kidney volume compared with the LP group [9]. However the long-term consequences of these results should be further evaluated.

Lastly it has been recently published that protein content of infant formulas may influence not only the growth pattern in the first two years, but also the risk of developing obesity at school-age, showing a 2.43 increased risk of obesity at 6 years of age in the HP vs LP group [10].

In conclusion these results suggest that early nutritional intervention programs are needed to avoid negative long-term consequences on health, especially to prevent “non-communicable diseases”in adult age.
